# Optimization strategies of mesenchymal stem cell-based therapy for acute kidney injury

**DOI:** 10.1186/s13287-023-03351-2

**Published:** 2023-04-30

**Authors:** Zhangning Fu, Yifan Zhang, Xiaodong Geng, Kun Chi, Chao Liu, Chengcheng Song, Guangyan Cai, Xiangmei Chen, Quan Hong

**Affiliations:** 1 Medical School of Chinese PLA, Beijing, China; 2 Department of Nephrology, First Medical Center of Chinese PLA General Hospital, Nephrology Institute of the Chinese PLA, State Key Laboratory of Kidney Diseases, National Clinical Research Center for Kidney Diseases, Beijing Key Laboratory of Kidney Disease Research, Beijing, China; 3 Beidaihe Rehabilitation and Recuperation Center, Chinese People’s Liberation Army Joint Logistics Support Force, Qinhuangdao, China; 4 Department of Critical Care Medicine, First Medical Center of Chinese PLA General Hospital, Beijing, China; 5 Department of Nephrology, Beijing Electric Power Hospital, Beijing, China

**Keywords:** Mesenchymal stem cells, Local administration, Three-dimensional culture, Preconditioning, Acute kidney injury

## Abstract

Considering the high prevalence and the lack of targeted pharmacological management of acute kidney injury (AKI), the search for new therapeutic approaches for it is in urgent demand. Mesenchymal stem cells (MSCs) have been increasingly recognized as a promising candidate for the treatment of AKI. However, clinical translation of MSCs-based therapies is hindered due to the poor retention and survival rates as well as the impaired paracrine ability of MSCs post-delivery. To address these issues, a series of strategies including local administration, three-dimensional culture, and preconditioning have been applied. Owing to the emergence and development of these novel biotechnologies, the effectiveness of MSCs in experimental AKI models is greatly improved. Here, we summarize the different approaches suggested to optimize the efficacy of MSCs therapy, aiming at promoting the therapeutic effects of MSCs on AKI patients.

## Introduction

Acute kidney injury (AKI), defined as a rapid increase in serum creatinine, decrease in urine output, or both, is a common clinical syndrome caused by multiple factors, including renal ischemia, sepsis, toxic effects from drugs, and pigment-related injury from myoglobin or hemoglobin [1, 2]. According to a recent statistical report, AKI occurs in approximately 13.3 million people per year worldwide and the number is still increasing [1, 3]. Except for several acute symptoms, AKI is strongly associated with subsequent chronic kidney disease (CKD) and end-stage kidney disease (ESKD) requiring necessary renal replacement therapy (RRT) or transplantation [4–6]. These sequelae place a significant financial burden not only on the patients and their families but also on the public healthcare services [7]. Current therapeutic approaches for AKI remain predominantly supportive and preventive, lacking in targeted pharmacological management [8]. In addition, since AKI often coexists with other syndromes such as heart failure, liver failure, and sepsis, AKI patients usually receive concomitant medications [1, 9], which may consequently result in the increased risk of adverse effects. It is therefore imperative to develop more safe and effective strategies to treat and prevent the progression of AKI.

Mesenchymal stem cells (MSCs) are a kind of cells with robust self-renewal and multi-lineage differentiation potential, existing in many tissues including bone marrow, adipose tissue, umbilical cord blood, and placenta [10]. MSCs secret diverse cytokines, chemokines, growth factors, exosomes, and microvesicles (MVs), which exert cell proliferative, anti-fibrotic, anti-inflammatory, anti-apoptosis, angiogenic, regenerative, and immunomodulatory effects [11–14]. Besides the release of paracrine or endocrine factors, the therapeutic mechanism of MSCs therapy also involves direct cell-to-cell interactions [15]. Increasing evidence has shown the promising renal protective effects of MSCs in AKI [16–20]. However, there are still some limitations hindering the clinical success of this MSCs-based therapy, for example, the low engraftment, poor survival, and the impaired paracrine ability of MSCs after administration [21]. To overcome these obstacles, many innovative approaches have been explored in recent years. This review discusses these novel strategies in the setting of AKI.

## Strategies for improving the therapeutic effects of MSCs

Advances in MSCs biology and bioengineering have shed light on new strategies that have the potential to address many of the limitations related to MSCs-based therapy. These strategies include the improvement in administration routes, the application of three-dimensional (3D) approaches, and the use of preconditioning methods.

### Improvement in administration routes

**Fig. 1:**
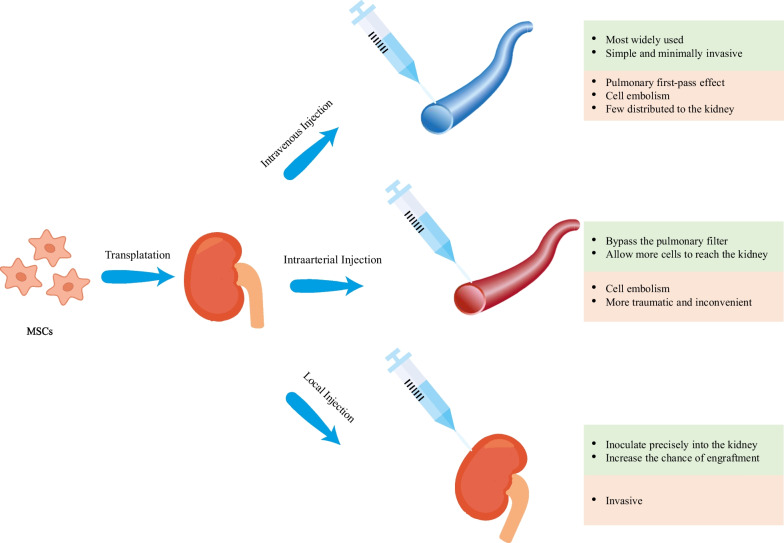
Common administration routes for MSCs transplantation in AKI models

For MSCs to exert their multiple therapeutic functions, a sufficient number of cells are required to be transferred to the sites of injury, which is the basis of MSCs treatment. Therefore, the choice of route of administration appears to be one of the most critical factors influencing the efficacy of MSCs-based therapy. However, no consensus has been reached on the best route for the administration of MSCs. For current preclinical and clinical trials, intravenous, intra-arterial and topical are the three commonly used routes (Fig. 1) [22].

Intravenous (IV) administration is the most widely used route in recent studies [22]. This route is a simple and minimally invasive way to deliver MSCs systematically into the animal models and human bodies. Despite these benefits, there is a great potential for MSCs to be trapped in the lungs, which is known as the pulmonary first-pass effect [23, 24]. In addition to the lungs, liver is also indicated to be a concentrated gathering place of MSCs after IV injection [25], resulting in an inadequate therapeutic concentration in the kidney. Investigators have attempted to employ a higher dose of MSCs to solve this problem but this may increase the risk of adverse events such as pulmonary embolism and thrombotic complications like sinusoidal obstructive syndrome [26–29]. Intra-arterial (IA) administration has been proved to be more efficacious than IV administration and has been used as an alternative in some treatment indications [22]. Selective IA delivery of MSCs can bypass the pulmonary filter, improving the homing of MSCs and allowing more cells to reach the targeted tissue. However, this route also has the limitation of entailing the risk of cell embolism and is more traumatic as well as inconvenient in clinical practice [22, 30]. Local administration of MSCs is more advantageous compared with IV and IA delivery and thus has become a new focus of current research [31]. MSCs can be inoculated precisely into the kidney via this route, increasing the chance of engraftment and enhancing their therapeutic potential. In addition, local injection avoids the lung barrier, decreasing the risk of lung infarction and mortality [24, 30]. This injection method may be especially beneficial for kidney regeneration as it allows vast amounts of cells to be located at the site of interests. Several local delivery methods have been tested in the animal models of AKI and have shown encouraging results in recent years [31–43] (Table 1). Among them, renal cortex injection is more widely researched (Table 1), possibly because of its relatively strong operability and reversible injury to the kidney [44]. As shown in Table 1, renal function recovery has been observed and the tubular injury has been ameliorated, with no identifiable safety concerns. However, since few studies have directly compared these different routes of MSC transplantation, additional research is needed to further explore the optimal routes of MSCs administration.

**Table 1:** Summary of the local delivery methods of MSCs in AKI models

References	Year	Animal	AKI model	MSCs source	Injection route	Renal outcomes
Huang et al. [31]	2022	Mice	I/R	UC-MSCs	Subcapsular / Parenchymal	Improvement in renal function and tubular repair; Reduction in tubular injury and fibrosis
Fu et al. [32]	2022	Mice	Glycerol-induced	BM-MSCs	Artificial kidney capsule packed with MSCs	Enhancement of renal function; Attenuation of tubular injury and fibrosis
Yang et al. [33]	2021	Mice	AAN	BM-MSCs	Ultrasound-guided injection of MSCs into the greater omentum	Improvement in renal function; Amelioration of tubular necrosis, peritubular interstitial fibrosis and inflammation
Wang et al. [34]	2020	Mice	I/R	HP-MSCs	Subcortical	Recovery of renal function; Facilitation of angiogenesis; Decrease in renal fibrosis
Paglione et al. [35]	2020	Rats	I/R	HO-MSCs	Parenchymal	Acceleration of renal functional recovery; Amelioration of tubular injury
Havakhah et al. [36]	2018	Rats	I/R	BM-MSCs	Parenchymal	Increase in renal function
Huang et al. [37]	2017	Rats	I/R	AD-MSCs	Subcortical	Improvement in renal function; Promotion of vascularization; Reduction in tissue injury and apoptosis
Geng et al. [38]	2017	Mice	Glycerol-induced	BM-MSCs	Biological membrane packed with MSCs	Increase in renal function; Decrease in renal tubular lesions and apoptosis
Feng et al. [39]	2016	Mice	I/R	AD-MSCs	Subcortical	Improvement in renal function; Enhancement of anti-inflammatory effects
Zhang et al. [40]	2014	Rats	I/R	AD-MSCs	Subcortical	Improvement in renal function, vascularization, apoptosis and histological injury
Cheng et al. [41]	2013	Mice	Cisplatin-induced	BM-MSCs	Subcapsular	Improvement in renal function; Reduction in tubular injury and cast formation
Gao et al. [42]	2012	Rats	I/R	AD-MSCs	Subcortical	Improvement in renal function, microvessel density and tubular cell proliferation
La Manna et al. [43]	2011	Rats	I/R	FM-MSCs	Subcortical	Acceleration of renal functional recovery; Amelioration of tubular injury and inflammation

*AAN* aristolochic acid nephropathy, *AD-MSCs* adipose-derived mesenchymal stem cells, *AKI* acute kidney injury, *BM-MSCs* bone marrow-derived mesenchymal stem cells, *FM-MSCs* fetal membrane-derived MSCs, *HO-MSCs* human omental-derived mesenchymal stem cells, *HP-MSCs* human placenta-derived mesenchymal stem cells, *I/R* ischemia/reperfusion, *MSCs* mesenchymal stem cells, *UC-MSCs* umbilical cord-derived mesenchymal stem cells

### 3D stem cell approaches for both culture and delivery

**Fig. 2:**
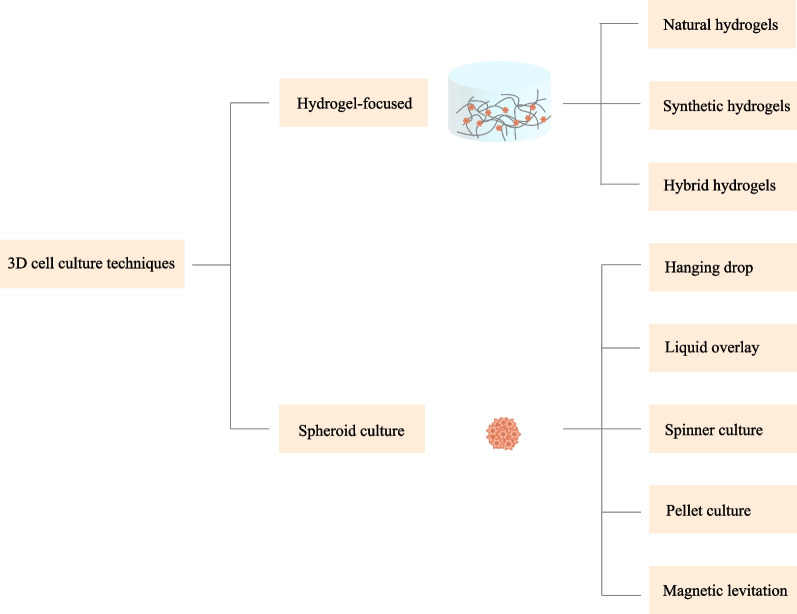
Technologies applied in 3D cell culture

Aside from the delivery mode, several other factors such as the cell growth environment also impact the retention and viability of the stem cells [21, 45]. Conventional approaches for MSCs culture are based on two-dimensional (2D) systems. Cells culture on these platforms often quickly undergoes senescence and a loss of cell functions [46–48]. To address these drawbacks, a beneficial 3D microenvironment is necessary to be designed to bridge the gap between the traditional culture system and the complex architecture in vivo. In addition, it is also worth noting that 3D approaches have become a frontier in stem cell delivery by virtue of their superior capability of promoting the survival and function of the transplanted cells [49]. Various methods have been developed to meet the demand for 3D cell culture and delivery over recent years. Broadly, these approaches can be categorized into scaffold-based (hydrogel-focused) and scaffold-free (spheroid culture) strategies (Fig. 2).

#### Hydrogel-focused strategies

Hydrogels have been considered as one of the most promising candidates for 3D stem cell culture and delivery [50, 51]. Hydrogels are chemically or physically cross-linked 3D porous polymeric networks characterized by high water content and tailorable mechanical, physical, and chemical properties [51–53]. The high moisture content and porous structure of hydrogels make it possible for the nutrients and metabolites to be transported in the networks [54, 55]. Hydrogels in this way, can act as an artificial extracellular matrix (ECM) surrounding the cells, providing necessary conditions for cell–cell and cell–matrix interactions, which as a result influence the behaviors and functions of MSCs [21]. To date, a large number of hydrogels have been investigated to mimic the native microenvironment where the cells reside in vivo, ranging from natural to synthetic origin [21]. The utility of hydrogels as scaffolds in supporting the growth and function of the cells has also been demonstrated in many preclinical AKI models [32, 39, 42, 56]. Below, we outline the categories of hydrogels and their corresponding properties.

##### Natural polymer hydrogels

Natural polymer-based hydrogels have attracted considerable attention over the past decade due to their good biocompatibility, biodegradability, and environmental friendliness [57]. Widely used natural polymers include agarose [58], alginate [59], chitosan [60], hyaluronic acid [61], collagen [62], gelatin [63], and fibrin [64], which can be further sub-classified into polysaccharide-based and protein-based hydrogels [48].

*Agarose* Agarose is a prominent marine polysaccharide extracted from agar or red seaweeds [58], possessing great biocompatibility, tunable mechanical characteristics, non-toxicity, non-immunogenicity, and thermo-reversible gelling properties [65]. This gel is made up of alternating residues of 1,3-linked β-d-galactopyranose and 1,4-linked 3,6-anhydro-α-l-galactopyranose [66]. Research works have shown that agarose can be used as culture scaffolds to enhance cell attachment and proliferation [55]. In addition, its thermally reversible characteristics offer great opportunities for its injection into the kidney with minimal invasiveness. However, agarose may be related to less enhanced cell functionality [65]. As agarose is seldom employed in current research of AKI, its applicability in renal tissues needs to be further studied.

*Alginate* The natural polymer alginate is a hydrophilic linear polysaccharide isolated from brown algae and certain bacteria, consisting of β-d-mannuronic acid (M) and α-l-guluronic acid (G) [59]. With the benign nature of cost-effectiveness, high biocompatibility, low cytotoxicity, and appropriate rheological properties, this soluble biopolymer is nowadays one of the most commonly employed bioinks in 3D bioprinting [67]. Prior to its use as a bioink, alginate has been extensively explored as a culture system and delivery vehicle for MSCs in the fields of regenerative medicine and tissue engineering [68–72]. Alginate also plays a significant role in the controlled release of the paracrine factors derived from MSCs [71, 73, 74]. However, alginate offers poor biodegradability and cell adhesive properties, which limit its potential applications [67, 70]. Investigators are trying to overcome these limitations, and a recent work has indicated that hydrogels composed of alginate reinforced with hyaluronic acid may be an exquisite candidate for AKI intervention [75].

*Chitosan* Chitosan, a linear polysaccharide composed of randomly dispersed β- [1–4]-linked d-glucosamine (deacetylated unit) and *N*-acetyl-d-glucosamine (acetylated unit), derived from chitin, is the second most abundant natural biopolymer available on earth [70]. Chitosan can be found in the exoskeleton of crustaceans and the cell envelope of fungi [60]. Aside from having structural similarity to glycosaminoglycans contained in the ECM, chitosan owns the features of biocompatibility, biodegradability, microbial resistance, non-toxicity and low cost [76]. This type of hydrogel is also sensitive to pH and temperature [65], which make it amenable to modification and be used in different kinds of conditions. Recent in vivo studies have highlighted the potency of chitosan-based hydrogels in improving the retention and survival as well as the therapeutic benefits of MSCs in AKI [39, 42]. Though seemingly quite promising, the poor mechanical strength and water solubility of chitosan under physiological conditions limit its use in biomedical applications. Conjugation with peptides or other hydrogels may assist to overcome some of these drawbacks [48, 65].

*Hyaluronic acid* Hyaluronic acid (HA) is a relatively high molecular weight non-sulfated glycosaminoglycan (GAG) containing repeated units of (β-1,4)-linked d-glucuronic acid and (β-1,3)-linked *N*-acetyl-d-glucosamine [77]. It is widely distributed throughout the body of adult mammals including connective tissue, synovial fluid, and vitreous humor [61, 78]. HA is an important component of ECM and is essential for cell growth, angiogenesis, embryonic development, wound healing, matrix organization, and morphogenesis [79]. HA has some remarkable properties such as non-adhesiveness, native bio-functionality, hydrophilia, and biodegradability [61, 65]. These advantages make hydrogels built from HA increasingly versatile for a myriad of biomedical applications. Evidence has shown that HA hydrogels can facilitate cell migration and adhesion by binding to the transmembrane receptor CD44 [80]. The implantation of stem cells into the HA hydrogels affects the release of cytokines/chemokines, counterbalancing the secretion of proinflammatory mediators from the immune cells, thereby influencing the immune response and ameliorating the renal damage [81]. Moreover, the highly reversible thermal properties of the HA hydrogels offer great conditions for their use as an injectable scaffold for the culture and delivery of MSCs or an implant material for the repair and reconstruction of the soft and hard tissues [82]. However, for the lack of stability at the body temperature and the ability of controlled release of the bioactive molecules, necessary methods such as chemical modification and covalent crosslink are needed to improve the performance of HA hydrogels [83].

*Collagen* Collagen, a fiber-like structure, is the most abundant structural protein present in the mammalian ECM [62]. Collagen exhibits a unique triple-helical structure with a repeating amino acid sequence (Gly-X–Y)n [84]. It can be easily manipulated through chemical and physical cross-linking or by blending with other polymers [85]. Owing to its biocompatibility, biodegradability, elasticity as well as structural similarity to the tissues, collagen hydrogels have been frequently investigated as a biomimetic 3D culture scaffold to support cell growth [70]. Previous studies have demonstrated that collagen-based scaffolds can enhance cell retention, cell functionality, cell proliferation, and phenotype maintenance, which thereby increases the therapeutic effects of MSCs for AKI [31, 37, 70]. Nevertheless, pure collagen scaffolds have weak structural stability and mechanical strength [86]. Recent advances in scaffold formulation have contributed to the improvement in the collagen-based hydrogel system.

*Matrigel* Matrigel, a basement–membrane matrix generated from Engelbreth–Holm–Swarm (EHS) mouse sarcomas, is a widely used collagen-containing hydrogel in tissue engineering applications [87]. The primary components of Matrigel are laminin, collagen IV, entactin, and the heparin sulfate proteoglycan [88]. Matrigel also contains a series of growth factors such as transforming growth factor (TGF) family peptides, fibroblast growth factors (FGFs), and insulin-like growth factors (IGFs), as well as enzymes [89]. Collectively, these components contribute to the excellent biological function of Matrigel. Although Matrigel has been tested as a cell culture tool for several decades [88], its applicability is severely limited due to its ill-defined, complex, and variable constituent [90]. The undefined compositions and antigenicity of Matrigel may lead to batch-to-batch differences in mechanical and biochemical characteristics in cell culture experiments, making it hard to characterize cell behavior and reproduce, which are major hurdles in fundamental research [87].

*ECM* Kidney ECM hydrogels, obtained through decellularization process, have attracted substantial attention in recent years as new solutions to kidney injuries [91]. By removing the cellular components and retaining the proteins, glycosaminoglycans, as well as growth factors present in the native tissue, these hydrogels are nonimmunogenic, biocompatible, and biologically active [92, 93]. Notably, in contrast to hydrogels composed of individual ECM components, kidney ECM hydrogels reserve the full biochemical complexity of the kidney tissue and, unlike Matrigel, do not consist of proteins originated from tumorigenic cells [94]. Kidney ECM hydrogels are currently being evaluated as an injectable scaffold to facilitate the repair and reconstruction of the renal tissue, and the results are encouraging [91, 95]. Nevertheless, like all natural materials, the properties of kidney ECM hydrogels and the effects of these properties upon cell behaviors are neither well understood nor controlled [94]. Future studies should further elucidate these issues, providing further insight into the management of AKI.

*Gelatin* As a hydrolytic product of natural collagen, typically of bovine or porcine origin, gelatin is a biocompatible and biodegradable polypeptide containing 18 different kinds of amino acids [70, 96]. In contrast to collagen, gelatin does not elicit any noticeable antigenicity under physiological conditions [96]. In addition to the above advantages, gelatin also has some other desirable properties such as commercial availability, cost economy, water solubility, adhesiveness, and easy processability, making it attractive in the applications of biomedicine [63]. MSCs-laden gelatin-based hydrogels have been shown to prolong the survival of MSCs and thus promote the repair of injured tissues in experimental AKI models [32]. Still, there are disadvantages existed in gelatin hydrogels, including poor mechanical strength, rapid enzymatic degradation, and inferior heat stability [97]. It is well known that pure gelatin has a solgel transition point around body temperature [98]. Therefore, pure gelatin could be injected as a low-viscosity fluid at 37 °C, but failed to form a stable hydrogel in vivo. Further modification is required to help improve the overall properties of native gelatin.

*Fibrin* Fibrin is a kind of natural polymer derived from key proteins involved in the blood clotting process [64]. In other words, it comes from fibrinogen and thrombin. The morphology, mechanical properties, and stability of fibrin hydrogels can be easily modulated by controlling the ratio of fibrinogen and thrombin in the hydrogels [99]. Fibrin-based hydrogels have been widely utilized for culturing and delivering MSCs due to their unique viscoelastic behavior, biocompatibility, biodegradability, and hemostasis [100–102]. When used as cell delivery systems, fibrin hydrogels have the advantage of being able to be implanted through injection without invasive surgery [51]. However, fibrin hydrogels face the challenges of weak mechanical strength and fast degradation speed, which limit their applications in renal diseases [48]. Researchers have tried to combine fibrin with other molecules or biomaterials to enhance its inherent biological properties. The usability and validity of these fibrin-based hydrogels in AKI have yet to be clarified.

##### Synthetic polymer hydrogels

Synthetic hydrogels are constructed with industrially manufactured polymers. Unlike natural hydrogels, synthetic hydrogels provide researchers with highly versatile materials that can be precisely controlled and designed [103, 104]. In addition, many synthetic hydrogels are essentially bioinert, allowing engineers to specifically modulate the cell–material interactions [77]. In this perspective, more predictable results can be achieved. Poly (ethylene glycol) (PEG) is the most widely implemented bioinert synthetic polymer in 3D cell culture [103]. Other commonly used synthetic hydrogels include poly (vinyl alcohol) (PVA) and poly [2-hydroxyethyl methacrylate] (PHEMA) [103].

*Poly (ethylene glycol) (PEG)* Poly (ethylene glycol) (PEG), sometimes referred to as poly (ethylene oxide) (PEO) depending on its molecular weight, is a very popular synthetic hydrophilic polymer used for hydrogel formation [85]. The basic structure of PEG is PEG diol with hydroxyl groups at each terminus, which can be converted into other functional groups like methyloxyl, carboxyl, amine, thiol, azide, vinyl sulfone, acetylene, and acrylate [105]. PEG has been considered as an ideal candidate for cell culture due to its non-toxicity to living tissues, superior resistance to protein adsorption, and ease of modification [106]. Its relatively low protein absorption prevents undesired cell–matrix reactions on the one hand, while, on the other hand, it also precludes this kind of material from having any interactions with the cells, which plays an important instructive role in mediating cell growth and functions [107]. Consequently, this type of hydrogel needs to be further modified with peptides or proteins, allowing individual control over each property of the matrix. Although recent work has shown that PEG-based hydrogels could increase stem cell attachment and proliferation [108–110], few are applied in the treatment of AKI. Thus, additional studies are required to further evaluate their effect on the kidneys.

*Poly (vinyl alcohol) (PVA)* Poly (vinyl alcohol) (PVA) is a water-soluble semicrystalline synthetic polymer with a backbone composed only of carbon atoms [111]. PVA is also a type of protein-resistant hydrogel and offers great flexibility in terms of precursor design [112]. PVA has received great attention in biomedical fields because of its advantages such as biodegradability, non-toxicity, non-carcinogenicity, and excellent mechanical properties [113–115]. Although some previous studies have indicated the facilitating effect of PVA on MSCs proliferation and its safety for in vivo use [111, 116, 117], evidence is still lacking in renal application.

*Poly [2-hydroxyethyl methacrylate] (PHEMA)* Poly [2-hydroxyethyl methacrylate] (PHEMA), one of the most important members of the methacrylate polymers, is the first successfully employed hydrogel in biological fields [118]. The presence of free hydroxyl group in PHEMA leads to the highly hydrophilic nature of this hydrogel, which facilitates the transportation of solutes and oxygen [119, 120]. This property in conjunction with other properties like cytocompatibility, non-toxicity, and ease of tuning makes hydrogel fabricated from PHEMA a fit candidate for biomedical use, especially for controlled drug release [120–122]. However, PHEMA is relatively weak in mechanical strength and is considered nonbiodegradable, limiting its application in vivo. In this regard, modifications have to be made by incorporating some enzymatically susceptible monomers or cross-linking agents into the PHEMA hydrogels [103]. Similar to PEG and PVA, although modified PHEMA hydrogels have been shown to promote the attachment, spread, and proliferation of MSCs, their effects on the kidney have not been validated yet [123, 124].

##### Hybrid hydrogels

Both natural and synthetic hydrogels have their own advantages and disadvantages. To overcome the inherent drawbacks of the traditional single-component hydrogels, researchers have been devoting efforts to combine multiple kinds of polymers to form hybrid hydrogels [125]. In this regard, hydrogels can be endowed with some particularly desirable characteristics to better mimic the native microenvironment. Generally, these combinations can be classified into three types: [1] a mixture of two or more ingredients that can form hydrogels alone; [2] a cocktail of the materials in which at least one of them cannot form hydrogels alone; and [3] a mix of the functional groups and the biomaterials. Recent studies [32, 34, 37, 39, 42, 81] have reported that hybrid hydrogels displayed wonderful potency in enhancing the engraftment as well as the survival of MSCs, thereby accelerating the renal functional recovery (Table 2). However, as the use of these hydrogels for AKI is relatively new and there is only a modest amount of data about their performance on the kidneys, further research regarding their in vivo efficacy and safety is still needed.

**Table 2:** Overview of the hybrid hydrogels applied in AKI models

References	Year	Animal	AKI model	MSCs source	Type of hydrogel	Outcomes
Fu et al. [32]	2022	Mice	Glycerol-induced	BM-MSCs	mTG-gelatin hydrogel	Improvement in renal function; Reduction in tubular injury and fibrosis
Wang et al. [34]	2020	Mice	I/R	HP-MSCs	β-IGF-1C hydrogel	Enhancement of cell engraftment; Recovery of renal function; Facilitation of angiogenesis; Decrease in renal fibrosis
Huang et al. [37]	2017	Rats	I/R	AD-MSCs	Co-gels consisting of collagen and decellularized vascular matrix	Increase in survival and paracrine effects of MSCs; Amelioration of renal function; Promotion of vascularization; Reduction in tissue injury and apoptosis
Feng et al. [39]	2016	Mice	I/R	AD-MSCs	Chitosan-IGF-1C hydrogel	Promotion of MSCs retention and survival; Improvement in angiogenesis and renal function; Enhancement of anti-inflammatory effects
Zullo et al. [81]	2015	Mice	LPS-induced	Renal MSCs	HA-hydrogel consisting of ProNectin	Alternation of macrophage secretome and polarization; Improvement in renal and vascular function
Gao et al. [42]	2012	Rats	I/R	AD-MSCs	Chitosan chloride hydrogel	Improvement in MSCs retention and survival; Enhancement of host renal cell proliferation; Attenuation of host renal cell apoptosis; Improvement in renal function, microvessel density and tubular cell proliferation

*AD-MSCs* adipose-derived mesenchymal stem cells, *AKI* acute kidney injury, *β-IGF-1C hydrogel* hybrid β-sheet peptide hydrogel consisting IGF-1C domain and D-Form peptide, *BM-MSCs* bone marrow-derived mesenchymal stem cells, *HA* hyaluronic acid, *HP-MSCs* human placenta-derived mesenchymal stem cells, *IGF-1C* C domain peptide of insulin-like growth factor-1, *I/R* ischemia/reperfusion, *LPS* lipopolysaccharide, *MSCs* mesenchymal stem cells, *mTG-gelatin hydrogel* microbial transglutaminase enzyme-cross-linked gelatin hydrogel

#### Spheroid culture

Spheroid culture is also a promising method for 3D cell culture. Through this method, 3D aggregations of MSCs and their secreted ECM could be obtained without the involvement of a scaffold mimicking the real tissues. Several techniques have been used for spheroid fabrication, including hanging drop, liquid overlay, spinner culture, pellet culture, and magnetic levitation [126, 127].

The hanging drop technique was the earliest described method used for spheroid fabrication [127]. In this method, cells gather at the bottom of the droplet and spontaneously aggregate to form spheroids. Hanging drop culture has many advantages such as controllable spheroid size and no need for professional equipment [126]. Alternatively, liquid overlay also enables cell aggregation and is suitable for large-scale production. It allows cells to grow in plates with substrates that limit cell adhesion. Typically, the non-adherent substrate is composed of agarose or PEG [128]. Another popular method for spheroid formation is spinner culture. In this system, cell suspension is put into a flask which is continuously stirred. This approach is especially amenable for long-term culture and intensive cell expansion in addition to mass production [129]. Spheroids can also be generated by centrifugation, which is often referred to as the method of pellet culture. This method is commonly used to induce the differentiation of MSCs [126]. A more recently developed technique for spheroid culture is magnetic levitation. The resultant spheroids can be easily manipulated and tracked via this means [130]. In general, with the help of 3D spheroid culture, MSCs could better maintain their distinct phenotypic and functional properties as well as secrete higher levels of cytokines or other factors, which as a result, improves the therapeutic effects of MSCs for AKI [131, 132].

### Preconditioning methods

**Fig. 3:**
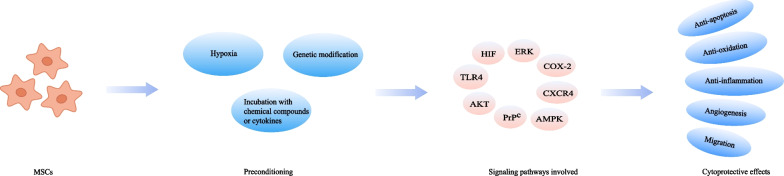
Preconditioning strategies for MSCs-based therapy in AKI models

Another critical bottleneck in the field of MSCs therapy is the harsh endogenous environment where the cells are located after transplantation [133]. This chokepoint has sparked the creation of preconditioning strategy (Fig. 3). Currently, researchers have attempted to pretreat MSCs with various physical, chemical, or biological factors to improve their efficacy in preclinical AKI models and the results are promising (Table 3).

**Table 3:** Preconditioning methods to enhance the effects of MSCs in AKI models

References	Year	Animal	AKI model	MSCs source	Type of preconditioning methods	Outcomes
Zhang et al. [40]	2014	Rats	I/R	AD-MSCs	Hypoxia	Increase in renal function; Improvement in anti-oxidative capacity, vascularization, apoptosis and histological injury;
Yu et al. [136]	2013	Rats	I/R	BM-MSCs	Hypoxia	Increase in MSCs migration, retention and paracrine capacity; Decrease in kidney injury; Enhancement of renal functional recovery
Liu et al. [137]	2012	Mice	I/R	BM-MSCs	Hypoxia	Increase in chemotaxis and viability of MSCs; Enhancement of secretion of proangiogenic and mitogenic factors; Improvement in renal function; Decrease in apoptotic cell death
Chen et al. [141]	2014	Rats	Sepsis-induced	AD-MSCs	Incubation with chemical compounds (melatonin)	Increase in renal function and antioxidants; Decrease in apoptosis, fibrosis, inflammation and kidney injury score
Cai et al. [144]	2014	Rats	I/R	BM-MSCs	Incubation with chemical compounds (atorvastatin)	Increase in the viability of MSCs; Improvement in renal function; Decrease in inflammation, tubular cell apoptosis and renal damage
Masoud et al. [145]	2012	Rats	I/R	BM-MSCs	Incubation with chemical compounds (SNAP)	Increase in MSCs survival, engraftment and proliferation; Decrease in apoptosis and fibrosis; Improvement in renal function
Tian et al. [146]	2012	Mice	I/R	NA	Incubation with chemical compounds (14S,21R-diHDHA)	Increase in secretion of HGF and IGF-1; Amelioration of renal function; Decrease in renal tubular cell death
La Manna et al. [43]	2011	Rats	I/R	FM-MSCs	Incubation with chemical compounds (HB)	Increase in renal function; Decrease in inflammation and histological injury
Mias et al. [142]	2008	Rats	I/R	BM-MSCs	Incubation with chemical compounds (melatonin)	Increase in MSCs survival and paracrine activity; Improvement in apoptosis, angiogenesis and renal function
Bai et al. [147]	2018	Mice	I/R	BM-MSCs	Incubation with cytokines (IL-17A)	Increase in secretion of PGE2; Decrease in inflammation and acute tubular necrosis score; Improvement in renal function
Xinaris et al. [148]	2013	Mice	Cisplatin-induced	BM-MSCs	Incubation with cytokines (IGF-1)	Increase in MSCs homing; Amelioration of renal structure impairment; Promotion of renal functional recovery
Ni et al. [150]	2021	Mice	Glycerol-induced	BM-MSCs	Genetic modification (Klotho)	Increase in secretion of VEGF, IGF-1 and HGF; Improvement in renal function
Roudkenar et al. [151]	2018	Rats	Cisplatin-induced	BM-MSCs	Genetic modification (Lcn2)	Increase in secretion of HGF, IGF-1, FGF and VEGF; Enhancement of renal function
Yan et al. [152]	2018	Mice	Sepsis-induced	NA	Genetic modification (HO-1)	Increase in renal function; Decrease in inflammation and acute tubular necrosis score
Liu et al. [153]	2018	Rats	I/R	BM-MSCs	Genetic modification (HO-1)	Increase in renal function; Decrease in acute tubular necrosis score
Mori da Cunha et al. [154]	2017	Rats	I/R	AF-MSCs	Genetic modification (VEGF)	Enhancement of the therapeutic effect of AF-MSCs through mitogenic, angiogenic and anti-inflammatory mechanisms
Zhaleh et al. [155]	2016	Rats	Glycerol-induced	BM-MSCs	Genetic modification (Nrf2)	Increase in renal function; Amelioration of kidney regeneration
Liu et al. [156]	2015	Rats	I/R	BM-MSCs	Genetic modification (HO-1)	Increase in renal function; Decrease in apoptosis
Mohammadzadeh‐Vardin et al. [157]	2015	Rats	Cisplatin-induced	BM-MSCs	Genetic modification (Nrf2)	Increase in renal function; Decrease in histological injury
Qi et al. [158]	2014	Mice	I/R	BM-MSCs	Genetic modification (survivin)	Increase in secretion of HGF and bFGF; Improvement in renal injury repair
Liu et al. [159]	2013	Mice	I/R	BM-MSCs	Genetic modification (CXCR4)	Increase in MSCs homing and paracrine capacity; Decrease in acute tubular necrosis score; Improvement in renal function
Yuan et al. [160]	2011	Mice	Cisplatin-induced	EMSCs	Genetic modification (VEGF)	Increase in anti-apoptosis effects; Improvement in cell proliferation, microcirculation, renal function and tubular structure
Chen et al. [161]	2011	Rats	I/R	UC-MSCs	Genetic modification (HGF)	Increase in renal function; Decrease in apoptosis and inflammation
Hagiwara et al. [162]	2008	Rats	I/R	BM-MSCs	Genetic modification (kallikrein)	Increase in renal function; Decrease in apoptosis and inflammation
Togel et al. [163]	2007	Mice	I/R	BM-MSCs	Genetic modification (hPAP)	Increase in vasculogenic capacity; Enhancement of renal recovery; Decrease in apoptosis

*AD-MSCs* adipose-derived mesenchymal stem cells, *AF-MSCs* human amniotic fluid derived mesenchymal stem cells, *AKI* acute kidney injury, *BM-MSCs* bone marrow-derived mesenchymal stem cells, *bFGF* basic fibroblast growth factor, *CXCR4* chemokine (C‐X‐C motif) receptor 4, *EMSCs* embryonic mesenchymal stem cells, *FGF* fibroblast growth factor, *FM-MSCs* fetal membrane-derived mesenchymal stem cells, *HB* hyaluronan monoesters with butyric acid, *HGF* hepatocyte growth factor, *HO-1* heme oxygenase-1, *hPAP* human placental alkaline phosphatase, *IGF-1* insulin growth factor-1, *IL-17A* interleukin-17A, *HP-MSCs* human placenta-derived mesenchymal stem cells, *IGF-1C* C domain peptide of insulin-like growth factor-1, *I/R* ischemia/reperfusion, *Lcn2* lipocalin-2, LPS lipopolysaccharide, *MSCs* mesenchymal stem cells; mTG-gelatin hydrogel microbial transglutaminase enzyme-cross-linked gelatin hydrogel, *NA* not available, *Nrf2* nuclear factor E2-related factor 2, *PGE2* prostaglandin E2, *SNAP* S-nitroso *N*-acetyl penicillamine, *US-MSCs* UC-MSCs umbilical cord-derived mesenchymal stem cells, *VEGF* vascular endothelial growth factor, *14S,21R-diHDHA* 14S,21R-dihydroxy-docosa4Z,7Z,10Z,12E,16Z,19Z-hexaenoic acid

#### Hypoxia

Hypoxia preconditioning has been frequently applied to improve the therapeutic potential of MSCs. MSCs are cultured in the environment with a 21% oxygen level generally, but once transplanted into the injured tissues, they often encounter hypoxic conditions with oxygen concentrations ranging from 1 to 6% [134]. The changed culture oxygen tension could affect a wide variety of cellular activities, including proliferation, differentiation, senescence, and metabolism, which may consequently compromise the cell ability for repairing dysfunctional organs [135]. Pre-exposure of MSCs to hypoxia could help conquer this obstacle. As reported in previous studies, hypoxic pretreated MSCs remarkably accelerate the functional and histological recovery in ischemic AKI models [40, 136, 137], which hypoxia-inducible factor-1α (HIF-1α) is thought to play a crucial role in this process [138].

#### Incubation with chemical compounds or cytokines

Pre-incubation of MSCs with various chemical compounds or cytokines has also been proved as an effective tool to improve the therapeutic efficacy of MSCs. Currently documented chemicals and biomacromolecules used for MSCs pretreatment in preclinical AKI models include melatonin, atorvastatin, and insulin growth factor-1 (IGF-1) (Table 3).

Melatonin is a neurohormone secreted by the pineal gland, having a variety of functions such as circadian rhythms regulation, anti-inflammation, and anti-oxidation [139, 140]. It was documented that MSCs pretreated with melatonin led to an enhanced therapeutic outcome in AKI models. The underlying mechanism might be that melatonin could suppress reactive oxygen species (ROS) generation and oxidative stress in either a receptor-dependent manner through ERK1/2, AMPK/ACC, and PrP^C^/PINK1 signaling pathways, or receptor-independent manner [141, 142]. The HMG-CoA reductase inhibitor atorvastatin has also been tested for treating AKI because of its anti-apoptotic, antioxidant, and anti-inflammatory effects [143]. Incubation of MSCs with atorvastatin prior to transplantation increased the viability of MSCs, resulting in the promotion of renal recovery. HMGB1/TLR4 pathway is considered to play a pivotal role during this process [144]. Another potential cell protective reagent is S‐nitroso N‐acetyl penicillamine (SNAP). It is a nitric oxide (NO) donor with the ability to regulate hemodynamics. In a model of renal ischemia/reperfusion (I/R) injury, MSCs preconditioned with SNAP were found more effective than those untreated, which was accompanied by an increase in the expression of PI3K/AKT pathway-related proteins [145]. 14S,21Rdihydroxy-docosa4Z,7Z,10Z,12E,16Z,19Z-hexaenoic acid (14S,21R-diHDHA) is also a cytoprotective agent exerting its beneficial effects on MSCs via PI3K/AKT pathway. A study demonstrated that preconditioning of MSCs with 14S,21R-diHDHA was able to ameliorate renal dysfunction and renal histological injury [146]. Similarly, the administration of MSCs primed with hyaluronan monoesters with butyric acid (HB), a differentiating agent, helped decrease the level of inflammation, which consequently reinforced the effectiveness of MSCs-based treatment in ischemic AKI [43].

The interaction between cytokines and their receptors can activate signaling cascades relevant to cell survival, proliferation, and migration. Therefore, cytokines preconditioning may have an impact on the fate of MSCs in vitro and in vivo. Interleukin-17A (IL-17A) pretreatment protected MSCs from harmful immune response, which thereby consolidated the therapeutic utility of MSCs. This improved effect was proved to be due to the increase in Treg percentages through the COX-2/PGE2 pathway [147]. Likewise, preconditioning with IGF-1 enhanced the migration of MSCs, leading to an improvement in the therapeutically relevant effects. An overexpression of CXCR4 was observed in this preconditioning method, which was considered to be associated with the increased migratory capacity [148].

#### Genetic modification

Another approach employed to increase the therapeutic potency of MSCs is genetic manipulation. Recent data have indicated that several genes are related to the function of MSCs and thus could be targets for modification. For example, heme oxygenase-1 (HO-1) is highly correlated with anti-oxidative activity and vascular endothelial growth factor (VEGF) is responsible for angiogenesis [149]. By overexpressing these specific factors, the migration ability, vasculotropic action, as well as the anti-inflammatory and survival capacities of MSCs could be boosted, contributing to the better recovery of renal function. PI3K/Akt, MEK/ERK, and other signaling pathways are involved in this cytoprotective process [150–163]. Furthermore, it should also be mentioned that in clinical practice, the application of genetic modification needs to be more prudent as consistent activation of some specific genes might be a risk factor for stem cell-derived tumors.

## Conclusion and future perspective

In conclusion, MSCs hold a considerable promise for the treatment of AKI. Nevertheless, the major outcomes of MSCs therapy in clinical trials of AKI have fallen far short of the theoretical effects of MSCs in preclinical studies. Challenges remain with respect to the clinical translation of this stem cell-based therapy. To address these challenges, various regimens including local administration, 3D cell culture as well as preconditioning have been exploited. In addition, considering the heterogeneity among patients, it is also important to realize that “one-size-fits-all” approach is clinically outdated. The characteristics of the patients such as age, genetics, and overall health status should be taken into consideration when applying the aforementioned strategies. Further research focused on the optimization of MSCs-based therapy is still needed to achieve the maximum therapeutic efficiency of MSCs in AKI patients.

## Data Availability

Not applicable.

## Abbreviations

AKI: Acute kidney injury
CKD: Chronic kidney disease
ECM: Extracellular matrix
ESKD: End-stage kidney disease
HA: Hyaluronic acid
HB: Hyaluronan monoesters with butyric acid
HO-1: Heme oxygenase-1
IA: Intra-arterial
IGF-1: Insulin growth factor-1
IL-17A: Interleukin-17A
I/R: Ischemia/reperfusion
IV: Intravenous
MSCs: Mesenchymal stem cells
MVs: Microvesicles
NO: Nitric oxide
PEG: Poly(ethylene glycol)
PHEMA: Poly(2-hydroxyethyl methacrylate)
PVA: Poly(vinyl alcohol)
ROS: Reactive oxygen species
RRT: Renal replacement therapy
SNAP: S‐nitroso *N*‐acetyl penicillamine
VEGF: Vascular endothelial growth factor
14S,21R-diHDHA: 14S,21Rdihydroxy-docosa4Z,7Z,10Z,12E,16Z,19Z-hexaenoic acid
2D: Two-dimensional
3D: Three-dimensional
